# Ibuprofen-Loaded Hyaluronic Acid Nanofibrous Membranes for Prevention of Postoperative Tendon Adhesion through Reduction of Inflammation

**DOI:** 10.3390/ijms20205038

**Published:** 2019-10-11

**Authors:** Chien-Tzung Chen, Chih-Hao Chen, Chialin Sheu, Jyh-Ping Chen

**Affiliations:** 1 Department of Plastic and Reconstructive Surgery and Craniofacial Research Center, Chang Gung Memorial Hospital at Linkou, Chang Gung University, Collage of Medicine, Kwei-San, Taoyuan 33305, Taiwan; ctchenap@cgmh.org.tw (C.-T.C.); chchen5027@gmail.com (C.-H.C.); 2 Department of Plastic and Reconstructive Surgery, Chang Gung Memorial Hospital at Keelung, Chang Gung University, College of Medicine, Keelung 20401, Taiwan; 3 Department of Chemical and Materials Engineering, Chang Gung University, Kwei-San, Taoyuan 33302, Taiwan; chialinsheu@gmail.com; 4 Research Center for Food and Cosmetic Safety, Research Center for Chinese Herbal Medicine, College of Human Ecology, Chang Gung University of Science and Technology, Taoyuan 33302, Taiwan; 5 Department of Materials Engineering, Ming Chi University of Technology, Tai-Shan, New Taipei City 24301, Taiwan

**Keywords:** nanofiber, ibuprofen, hyaluronic acid, post-operative adhesion, electrospinning, inflammation

## Abstract

A desirable multi-functional nanofibrous membrane (NFM) for prevention of postoperative tendon adhesion should be endowed with abilities to prevent fibroblast attachment and penetration and exert anti-inflammation effects. To meet this need, hyaluronic acid (HA)/ibuprofen (IBU) (HAI) NFMs were prepared by electrospinning, followed by dual ionic crosslinking with FeCl_3_ (HAIF NFMs) and covalent crosslinking with 1,4-butanediol diglycidyl ether (BDDE) to produce HAIFB NFMs. It is expected that the multi-functional NFMs will act as a physical barrier to prevent fibroblast penetration, HA will reduce fibroblast attachment and impart a lubrication effect for tendon gliding, while IBU will function as an anti-inflammation drug. For this purpose, we successfully fabricated HAIFB NFMs containing 20% (HAI20FB), 30% (HAI30FB), and 40% (HAI40FB) IBU and characterized their physico-chemical properties by scanning electron microscopy, Fourier transformed infrared spectroscopy, thermal gravimetric analysis, and mechanical testing. In vitro cell culture studies revealed that all NFMs except HAI40FB possessed excellent effects in preventing fibroblast attachment and penetration while preserving high biocompatibility without influencing cell proliferation. Although showing significant improvement in mechanical properties over other NFMs, the HAI40FB NFM exhibited cytotoxicity towards fibroblasts due to the higher percentage and concentration of IBU released form the membrane. In vivo studies in a rabbit flexor tendon rupture model demonstrated the efficacy of IBU-loaded NFMs (HAI30FB) over Seprafilm^®^ and NFMs without IBU (HAFB) in reducing local inflammation and preventing tendon adhesion based on gross observation, histological analyses, and biomechanical functional assays. We concluded that an HAI30FB NFM will act as a multi-functional barrier membrane to prevent peritendinous adhesion after tendon surgery.

## 1. Introduction

The limitation of hand function caused by postoperative tendon adhesion is mainly attributed to tendons adhering to surrounding tissues during the healing process, thereby limiting joint mobility [1]. Currently available anti-adhesion devices still exhibit certain problems. Seprafilm^®^ is an anti-adhesion membrane comprising hydrophilic hyaluronic acid (HA) and carboxymethyl cellulose. To prevent post-surgical adhesion after tendon surgery, Seprafilm^®^ shows some disadvantageous such as limited flexibility, poor handling capability, easily breaking into pieces, and a fast degradation rate [2,3]. SurgiWrap^®^ is a membrane that mainly comprises polylactic acid (PLA), which does not easily curl over to cover the whole surgical site. Furthermore, SurgiWrap^®^ is brittle due to the fact of its hydrophobic and nonporous natures, which also hinders nutrient exchanges in a surgical site with limited blood supply, such as during tendon repair operation, affecting postoperative healing [4]. Considering that an ideal anti-adhesion device requires properties such as mimicking the native tendon sheath, a nanofibrous membrane (NFM) obtained through electrospinning is expected to structurally serve as a tendon sheath to effectively separate damaged tendon tissue from surrounding tissues [5]. With the high porosity, suitable pore size, and excellent permeability associated with an NFM, it can also effectively prevent the penetration of fibroblasts without simultaneously compromising nutrient/waste exchanges [6].

Previous studies on anti-adhesion membranes to prevent tendon adhesion were mainly focused on electrospun nanofibers of HA-grafted poly(caprolactone) (PCL), poly(ethylene glycol) (PEG) blending with PCL, HA-loaded PCL, ibuprofen-loaded poly(lactic acid) (PLA), and ibuprofen-loaded PLA-PEG copolymer [6,7,8,9,10]. Incorporation of ibuprofen (IBU) in biodegradable electrospun NFMs is expected to inhibit inflammation [11]. With the anti-inflammatory property from IBU, a drug-loaded barrier membrane could augment its anti-adhesion efficacy just as nonsteroidal anti-inflammatory drugs (NSAIDs) such as IBU were reported to reduce flexion tendon adhesion [12,13]. Indeed, existing studies using nanomaterials for anti-adhesion purpose are based on biodegradable materials combined with other bioactive agents for better anti-adhesion outcomes [14,15]. For this purpose, anti-adhesion NFMs with multiple functions through combining with gene therapy or drugs conducive to healing should be developed [16,17,18]. In addition, as clinical Good Manufacture Practice (GMP)-grade electrospinning machines are already available commercially, the possibility of clinical applications of NFMs for anti-adhesion purposes may increase in the future [19].

Hyaluronic acid is a component of the extracellular matrix and is also present in the synovial fluid, making it biodegradable and biocompatible [20,21]. The main disadvantages of anti-adhesion products containing HA are their poor mechanical properties and fast degradation rates [22]. Poly(ethylene glycol) (PEG) (or poly(ethylene oxide), PEO) has favorable biocompatibility, as well as anti-adhesive, non-toxic, and anti-protein adsorption properties. Although the main mechanism of anti-adhesion property has yet to be clarified, the anti-protein adsorption mechanism of PEG is believed to be related to the exclusion effect of larger molecules in addition to favorable hydrophilicity [23,24,25,26]. Ibuprofen is a nonsteroidal drug with anti-inflammatory properties. Its absorption rate is not affected by age and it is metabolized in the liver with approximately 90% of metabolites being excreted through the urine [27,28]. This study aims to use iron ions (Fe^3+^) and 1,4-butanediol diglycidyl ether (BDDE) to prepare dual crosslinked IBU-loaded HA NFMs that could effectively prolong the degradation time and reduce cell attachment and penetration. It is expected that the drug-loaded NFMs may achieve better postoperative anti-adhesion effects from controlled released of IBU to reduce inflammatory reactions at the surgical site.

## 2. Results and Discussion

### 2.1. Characteristics of the NFMs

**Figure 1:**
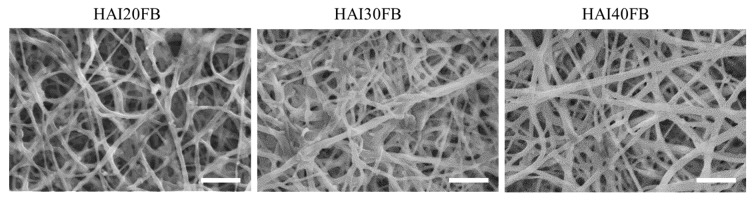
The SEM micrographs of HAI20FB, HAI30FB, and HAI40FB nanofibrous membranes (NFMs). Bar = 10 μm. HAIFB nanofibers containing 20, 30 or 40% IBU become HAI20FB, HAI30FB and HAI40FB NFMs, respectively.

As shown in Figure 1, SEM images revealed that electrospinning and dual crosslinking post-treatment could successfully prepare continuous IBU-loaded HAIFB nanofibers containing 20, 30 or 40% IBU [29]. The average fiber diameter increased with IBU, which were 0.52 ± 0.16 μm, 0.58 ± 0.17 μm, and 0.63 ± 0.21 μm for HAI20FB, HAI30FB, and HAI40FB, respectively. Nonetheless, no significant difference in fiber diameter was found among all NFMs.

**Figure 2:**
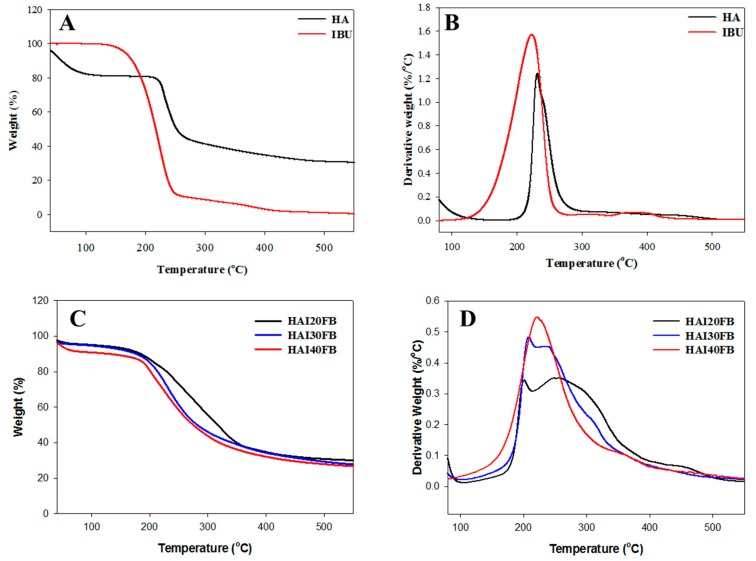
The TGA (**A**,**C**) and DTG (**B**,**D**) curves of hyaluronic acid (HA) and ibuprofen (IBU) (**A**,**B**) and HAI20FB, HAI30FB and HAI40FB NFMs (**C**,**D**).

The thermal properties of HAI20FB, HAI30FB, and HAI40FB NFMs were analyzed from the TGA and DTG curves in Figure 2. The TGA curves indicated substantial weight loss for all NFMs when temperature was increased to 600 °C, with the initial weight loss before 100 °C likely due to the water bound to HA. The thermal decomposition started at ~210 °C and gave rise to 31.0% residual weight for HA. In contrast, the onset of weight loss for IBU started earlier at ~130 °C with ~0% residual weight (Figure 2A) [30]. A broad DTG peak with a peak decomposition temperature occurring at ~220 °C was observed for IBU, while HA gave a sharper DTG peak with the maximum weight loss being observed at ~231 °C (Figure 2B). For NFMs, similar decomposition curves were observed from TGA, but the remaining weight at 600 °C decreased with higher IBU loading in the NFMs (Figure 2C). This trend could be explained from Figure 2A as IBU, but not HA, can be completely decomposed at higher temperatures. The DTG curves for NFMs showed a broad decomposition peak temperature due to the similarity of the peak temperatures of HA and IBU (Figure 2B) with a shoulder around 210 °C (Figure 2D). By increasing IBU content, this shoulder in the DTG curves diminished to result in only one discernible peak for HAI40FB.

**Figure 3:**
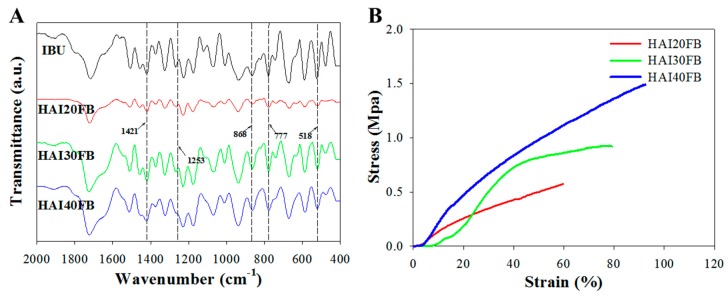
The FTIR spectra (**A**) and typical stress–strain curves (**B**) of the HAI20FB, HAI30FB, and HAI40FB NFMs.

From Figure 3A, the FTIR analysis of NFMs confirmed successful chemical crosslinking of HA by BDDE, where the peak observed at 1253 cm^−1^ corresponded to the newly generated C–O in ether or ester from the carboxyl groups and hydroxyl groups of HA [31]. The presence of IBU in the NFMs could be inferred from the appearance of peaks assigned to IBU in all HAIFB NFMs. The peak at 518 cm^−1^ denotes the aromatic C–H deformation of IBU whereas peaks at 777, 866, and 1421 cm^−1^ could be assigned to the CH_2_, CH, and CH–CO groups, respectively [32].

From mechanical testing, the NFMs showed typical stress–strain curves, as seen in Figure 3B, which indicated the ultimate tensile stress and tensile strain increased with increasing IBU loading in the membrane. Indeed, the ultimate stress (strain) of HAI40FB was 2.3 (1.5) times that of HAI20FB (Table 1). Similarly, the Young’s modulus also increased with IBU loading, with HAI40FB showing a significantly higher modulus than HAI20FB and HAI30FB. Without a significant increase in the fiber diameter with increasing IBU loading (Figure 1), the increase in the Young’s modulus and tensile stress may be related to more interactions between IBU and HA polymer chains, which could stabilize the crosslinked polymer networks [33]. Overall, the superior stretching capability and breaking strength will undoubtedly facilitate the application of the IBU-loaded NFMs to the curved and limited space around the operation site after tendon surgery.

### 2.2. Release of IBU from NFMs

**Figure 4:**
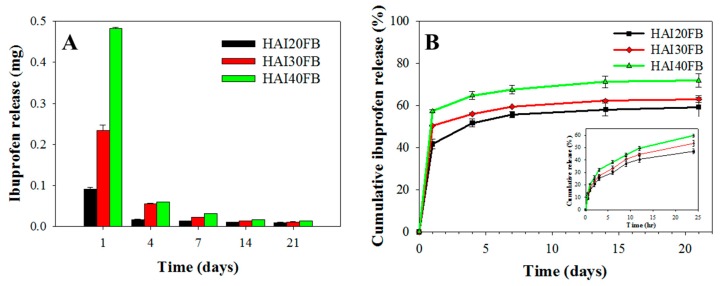
The released weight (**A**) and cumulative release percentage (**B**) of IBU from HAI20FB, HAI30FB, and HAI40FB NFMs. The insert in (**B**) is the release percentage of IBU within 24 h.

As shown in Figure 4A, the released IBU weight from the NFMs correlated with its loading percentage in the membranes. The released drug content diminished rapidly with time but continuous drug release could still be observed up to 21 days. The cumulative drug release showed a 55%, 59%, and 65% drug release percentage for HAI20FB, HAI30FB, and HAI40FB on day 7, respectively, which increased gradually to 59%, 62%, and 71% on day 21 (Figure 4B). Nonetheless, sustained release of IBU from all NFMs was observed during the first 24 h with 40%–60% of drug release after 24 h (Figure 4B, insert). In this study, the IBU-loaded NFM was prepared by crosslinking HA with BDDE from reaction of the carboxyl or hydroxyl groups of HA with the end groups of BDDE in ethanol. As IBU is a monocarboxylic acid, we cannot rule out the possibility that IBU could be covalently bound to the NFM during crosslinking. Therefore, we did not extract all IBU from the fibers to obtain the real content in the formulations but used the amount of IBU fed to the system upon preparation for calculation of the cumulative drug release in Figure 4B. The HAI40FB NFM therefore provided the fastest IBU drug release rate and the highest drug concentration for anti-inflammation efficacy. It should be noted that the burst drug release profile with massive release within a week will be critical to inhibit tissue adhesion [34], which is caused by an inflammatory response during the early stage of the tendon healing process. The fast release of IBU from the implanted NFMs may also contribute to alleviate the associated pain after tendon surgery [35].

### 2.3. In Vitro Cell Culture

#### 2.3.1. Cytotoxicity of NFMs

**Figure 5:**
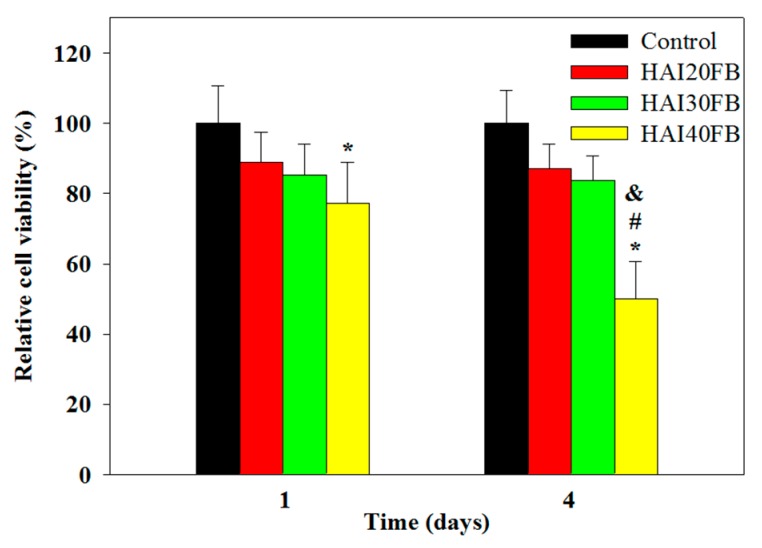
Cytotoxicity of NFMs by culture 3T3 fibroblasts with 24 h extracts (in cell culture medium) of HAI20FB, HAI30FB, and HAI40FB NFMs for 1 and 4 days. The relative cell viability was normalized with cells cultured in fresh cell culture medium (control), which was taken as 100%. * *p* < 0.05 compared with control; ^#^ *p* < 0.05 compared with HAI20FB; ^&^ *p* < 0.05 compared with HAI30FB.

As constituents of NFMs such as IBU or HA may have cytotoxic effect, the cytotoxicity of IBU-loaded NFMs was evaluated following the ISO standards after extracting the NFMs for 24 h with cell culture medium. The relative cell viability was based on the solution absorbance from the 3-(4,5-dimethylthiazol-2-yl)-5-(3-carboxymethoxyphenyl)-2-(4-sulfophenyl)-2H-tetrazolium (MTS) assay after normalizing with that of fresh culture medium (control) (Figure 5). The relative cell viability of HAI20FB and HAI30FB, but not HAIFB40, was not significantly different from the control on day 1. On day 4, the relative viability of cells grown in HAIFB40 extract was significantly less than all other groups and decreased from 77% to 50%, while still no significant difference was found for other NFMs from the control. The cytotoxicity found for HAIFB40 was in line with the faster IBU release rate and the higher concentration of released IBU at high IBU-loading shown in Figure 4. Indeed, previous reports indicated cell viability in the presence of IBU may depend on drug dosage and a higher concentration of IBU resulted in substantial cytotoxicity in vitro [36,37]. These results indicated that IBU may lead to toxic reactions in fibroblasts if in excess [38,39]. The consideration of NFM cytotoxicity pointed toward the choice of HAI30FB NFM to prevent tendon adhesion in vivo, which is biocompatible with the highest IBU loading.

#### 2.3.2. Penetration of Cells through NFMs

**Figure 6:**
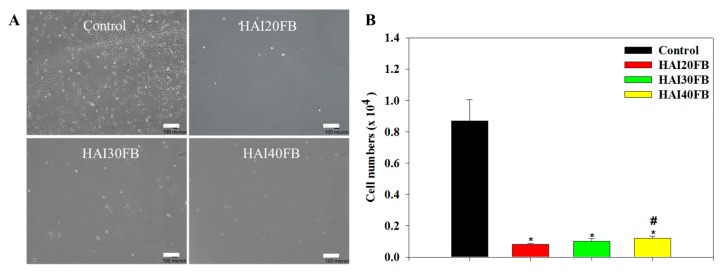
Migration of 3T3 fibroblasts through the control (no NFMs), HAI20FB, HAI30FB or HAI40FB NFMs in 24 h via optical microscopic observation of penetrated cells (**A**) and an assay of penetrated cell numbers (**B**). Bar = 100 μm. * *p* < 0.05 compared with control; ^#^ *p* < 0.05 compared with HAI20FB.

As exogenous healing occurred before endogenous healing after tendon surgery, tendon adhesion is likely to happen if no barrier is used to prevent the peripheral fibroblasts from invading. Considering the role of fibroblasts in adhesion formation, the design of an NFM should also consider the migration and penetration of fibroblasts through the barrier membrane at the surgical site. Toward this end, we studied 3T3 fibroblast cell penetration through an NFM using gradient serum concentration to drive cell migration. The control group without an NFM in a cell insert was compared with the NFM group where an NFM was closely fitted to the bottom of the cell insert. As the difference in serum concentrations among compartments in the culture chamber forced cell penetration through the cell inset, the extent of cell migration to the bottom chamber could be observed by an optical microscopy (Figure 6A). The microscopic observation of cells on the surface of the lower chamber revealed few cells could be seen in the NFM groups, while abundant cells were found in the control group. Follow-up experiments to quantify the viable cell number collected in the bottom chamber indicated the penetrated cell numbers were 0.10, 0.12, 0.14, and 0.87 × 10^4^ cells for HAI20FB, HAI30FB, HAI40FB, and the control groups, respectively (Figure 6B). Therefore, all NFMs could significantly reduce the number of cells that penetrated the membrane. That the number of cells which penetrated through the NFM was drastically different from and in the range of 11–16% of the control group indicated that the membrane could effectively block cell penetration. This drastic reduction in the number of cells that penetrated an NFM in vitro also suggested its efficacy to prevent tendon adhesion in vivo due to the extrinsic fibroblastic cells. Indeed, the dense fiber morphology and the macroporous structure of the NFM shown in Figure 1 facilitates its use as a physical barrier to prevent postoperative adhesion [40]. That the number of penetrated cells of HAI40FB was significantly higher than HAI20FB may be due to the difference in pore sizes of the membrane [41].

#### 2.3.3. Cell Attachment to NFMs

**Figure 7:**
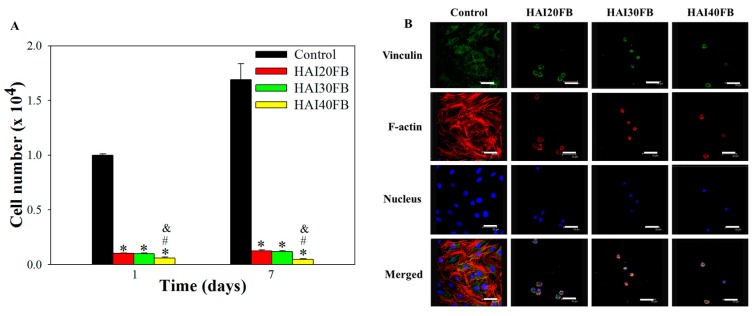
(**A**) Attachment and proliferation of 3T3 fibroblasts on TCPS (control) and HAI20FB, HAI30FB, and HAI40FB NFMs from assays of DNA contents. (**B**) The cytoskeletal arrangement and focal adhesion protein expression of 3T3 fibroblasts after cell seeding for 24 h were determined by actin cytoskeleton and vinculin staining and observed by confocal microscopy. Vinculin focal adhesion, actin cytoskeleton, and cell nucleus are shown in green, red, and blue, respectively. Bar = 50 mm. * *p* < 0.05 compared with control; ^#^ *p* < 0.05 compared with HAI20FB; ^&^ *p* < 0.05 compared with HAI30FB.

As fibroblast attachment is the first event responsible for adhesion formation, the adherence of fibroblasts to and cell proliferation on NFMs was compared with the control (TCPS) by seeding 3T3 cells directly on the membrane surface. The cell number after being cultured for 1 and 7 days was determined from DNA assays and shown in Figure 7A. The number of adhered cells on the TCPS (control) on day 1 was significantly higher than those on NFMs—up to 16.7 times, demonstrating that the membrane can effectively prevent cell attachment. There was no statistical difference between HAI20FB and HAI30FB, but HAI40FB showed a significantly lower adhered cell number. The drastic reduction of cell attachment to all NFMs on day 1 endorses the beneficial effects of IBU-loaded NFMs to reduce fibroblast adherence. During the process of adhesion formation, the deposition of fibroblasts and macrophages in the matrix led to the development of a fibrous band [42]. Therefore, as a critical step in adhesion formation, the examination of whether an NFM can reduce deposition of fibroblasts in vitro is important. All NFMs were observed to virtually eliminate fibroblast adherence and reduce the attached cell number by more than 90%, which strongly suggests an NFM can function in vivo to block fibroblast attachment to the injured surface, thus interrupting fibrin matrix formation that leads to adhesion formation [43]. We postulate the drastic drop in the number of adhered cells on NFMs may be correlated with the combined effect of IBU and HA in the membranes. Previously, dermal fibroblasts were found to show less attachment to HA-modified surfaces, which was explained based on the influence through the HA receptor CD44 [44]. By grafting HA to PCL NFMs, we also found less fibroblast adhesion compared to unmodified membrane surfaces [7]. Nonetheless, the significant reduction in the cell number for HAI40FB among all IBU-loaded NFMs may imply that the dosage of IBU influences cell adhesion as well, since a previous report suggested that IBU dosage directly leads to inhibition of cell adhesion [45].

Following the same trend as day 1, the TCPS group showed a distinctively higher cell number for all NFMs on day 7, while the HAI40FB NFM showed the minimum compared to all other groups. To compare the cell proliferation rate, the mean increase in cell number from day 1 to day 7 were calculated to be 1.69, 1.23, 1.19, and 0.74 for control (TCPS), HAI20FB, HAI30FB, and HAI40FB, respectively. The reduction in cell proliferation rate may be mainly due to the action of HA, which can inhibit the growth of fibroblasts [46]. Nonetheless, loading more IBU in NFMs (as for HAI40FB) may further induce cytotoxicity, which even reduces the cell number on day 7 from day 1. Taken together, increasing IBU loading in the membrane will influence cell proliferation, as evidenced from the reduced cell proliferation rate. Therefore, loading IBU up to 30% may be deemed as an optimized drug loading concentration without influencing the barrier and anti-adherence effects towards fibroblasts, which is expected to lead to successful tendon anti-adhesion in vivo.

Follow-up study to validate the molecular mechanism responsible for reduced cell attachment was carried out by examining the expression of a focal adhesion protein (vinculin) and cytoskeletal actin distribution in 3T3 fibroblasts under a confocal microscope (Figure 7B). The protein vinculin was labelled in green to visualize focal adhesion; the red F-actin protein was stained in red to confirm cytoskeletal arrangement; the cell nucleus was counterstained in blue. In the control group, fibroblasts attached to the TCPS surface exhibited a nearly flattened and spread morphology with a well-distributed fibrous F-actin cytoskeleton 24 h after cell seeding, while enhanced vinculin expression indicated increased cellular spreading. In comparison, cells attached to all NFMs showed restricted cytoskeletal distribution and minimal vinculin expression revealed from the rounded morphology. The circular shape of the cell indicated that the cell had extended to induce adhesion to a substrate. All NFMs showed similar cell spreading and cytoskeletal distribution characteristics with adhered cells, endorsing excellent cell adhesion inhibition behavior exerted by the membrane as shown from the quantitative cell attachment results in Figure 7A. Fewer cells were also found for the HAI40FB group compared to other NFMs, as seen in Figure 7A. Our previous results also supported reduced fibroblast adhesion to NFMs containing HA [47]. The cell penetration and attachment study thus confirmed the applicability of IBU-loaded NFMs for post-surgical anti-adhesion, other than HAI40FB due to the fact of its cytotoxicity. We therefore chose an NFM with the next highest IBU content (i.e., HAI30FB) for subsequent in vivo studies.

### 2.4. In Vivo Studies

#### 2.4.1. Gross Observation

**Figure 8:**
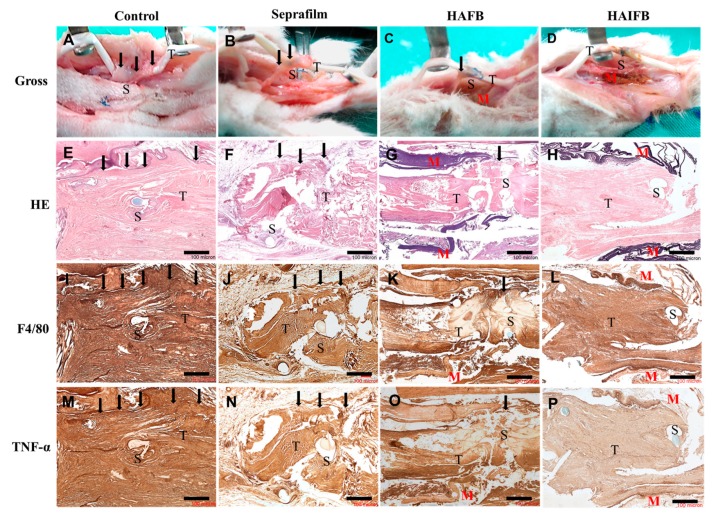
Gross view (**A**–**D**), H&E (**E**–**H**), F4/80 (**I**–**L**), and TNF-α (**M**–**P**) stainings of tissue sections of the repaired flexor digitorum profundus (FDP) tendons of the untreated control group and the experimental groups treated with Seprafilm^®^, HAFB, and HAIFB 3 weeks post-operation. Bar = 100 μm. T: tendon; S: suture; M: membrane; black arrows: sites where adhesion occurred.

From in vivo experiments, all animals were euthanized 3 weeks post-operation and gross view was adopted to observe tissue adhesion around the tendon repair site (Figure 8A–D). The animal study was divided into four groups, an untreated control group and three treatment groups using Seprafilm^®^, HAFB (without IBU) or HAIFB (with IBU, HAI30FB). The initial 3 week period was used for evaluation, as clinical rehabilitation in tendon healing is initiated and more critical during the early stage [34]. In the HAIFB group—NFMs with 30% IBU—the repaired tendon did not show adhesion with the surrounding tissues to exhibit a smooth morphology (Figure 8D). In comparison, tight peritendinous adhesions were noted at the repair site with the surrounding tissues in the untreated control group and strong force was required to separate them (Figure 8A). Moreover, the group treated with Seprafilm^®^ (Figure 8B) and HAFB (Figure 8C) showed some loose fibrous tissue connected to the tendon; therefore, considerable strength was still required to separate them from the surrounding tissues. Although moderate to mild tissue bridging was observed and adhesions could be removed by blunt dissection in both groups, the HAFB group showed relatively thinner adhesion bands. Based on gross observation, the extent of peritendinous adhesion was further evaluated and compared among four groups. The extent of tissue adhesion was graded into four categories with scores from 0 (no adhesion) to 3 (severe adhesion). The average adhesion scores of the control group, the group treated with Seprafilm^®^, HAFB, and HAI30FB were 2.67, 1.00, 0.83, and 0.17, respectively. The control group demonstrated the highest score, indicating the most severe adhesion. The HAI30FB group exhibited the lowest score, indicating its efficacy in preventing postoperative tendon adhesion (Figure 8D).

#### 2.4.2. Histological Staining

Histological analysis was performed to evaluate adhesion formation and inflammation around treated tendons by staining tissue slices with H&E and IHC staining of TNF-α and F4/80 (Figure 8E–P). In the control group, tendons were found to have tight adhesion to the surrounding tissues (Figure 8E) [48]. In the experimental groups treated with Seprafilm^®^ (Figure 8F) and HAFB (Figure 8G), a slight to moderate adhesion of the tendon to the surrounding tissues occurred [6]. In contrast, the HAIFB group displayed no adhesions between the surrounding tissues and the repaired tendon (Figure 8H). Unlike Seprafilm^®^, the NFMs displayed more physical stability in vivo as membrane remnants (indicated by M) could still be observed after 3 weeks. This provided the membrane with a continuous barrier effect to effectively prevent fibroblast invasion and adhesion formation with the surrounding tissues post-surgically.

The F4/80 is one of the surface receptors on macrophages, and when the tissue is injured, it is accompanied by an inflammatory reaction that causes macrophages to accumulate at the site of injury. The number of macrophages is tantamount to the expression of their surface receptors, which affects the F4/80 staining intensity [49]. From a microscopic point of view, the control group had the darkest F4/80 stained color, indicating the most serious inflammatory reaction (Figure 8I). Furthermore, the colors of the groups treated with Seprafilm^®^ (Figure 8J) and HAFB (Figure 8K) were observed to be darker than HAIFB (Figure 8L) in the vicinity of the wound, suggesting the number of macrophages was lowered by incorporating IBU in the NFM. This implies IBU released from the HAIFB NFM could inhibit the inflammatory reaction, thereby reducing the number of macrophages that accumulate at the tendon repair site. This observation undoubtedly endorsed that the anti-inflammatory action of HAIFB NFM during tendon healing may be correlated with its efficacy to prevent tendon adhesion [50].

Tumor necrosis factor alpha (TNF-α) is released by macrophages and activates proteins that stop apoptosis and promote cell proliferation and inflammation [51]; therefore, it is considered a critical factor in inflammatory responses. Tendon repair sites or adhesions all exhibited denser staining intensity for TNF-α in the control group (Figure 8M). This is because inflammation occurred at the site of injury, resulting in a larger aggregation of macrophages that release TNF-α, resulting in a darker color. The groups treated with Seprafilm^®^ and HAFB had the second darkest color (Figure 8N,O), indicating a notable inflammatory response [52]. Finally, the group treated with HAIFB had the lightest color (Figure 8P), confirming the membrane containing 30% IBU can reduce inflammation through drug release and simultaneously reduce the number of macrophages aggregated near the injured site [53].

#### 2.4.3. Evaluation of Tendon Adhesion by Biomechanical Evaluation

**Figure 9:**
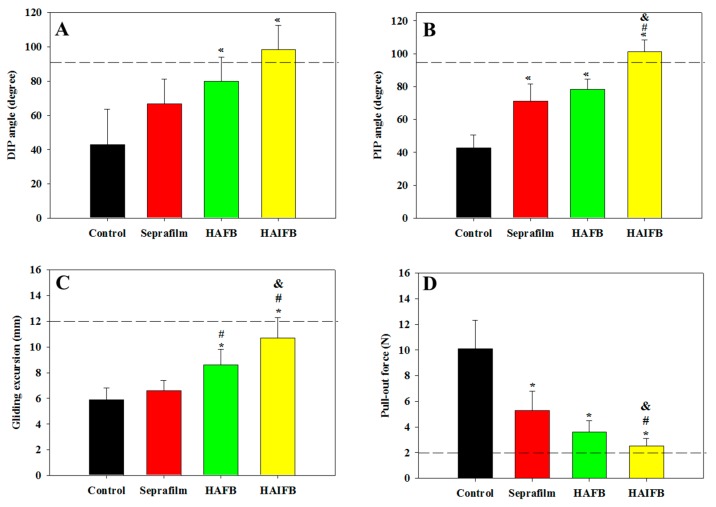
Evaluation of peritendinous adhesions from the distal interphalangeal (DIP) joint flexion angle (**A**), proximal interphalangeal (PIP) joint flexion angle (**B**), tendon gliding excursion (**C**), and pull-out force (**D**) 3 weeks post-operation. The dotted line represents the average value of the normal FDP tendon. * *p* < 0.05 compared with control, ^#^ *p* < 0.05 compared with Seprafilm^®^; ^&^ *p* < 0.05 compared with HAFB (*n* = 6 for each group).

Through biomechanical evaluation, including range-of-motion, gliding excursion, and pull-out force of the FDP tendons, the anti-adhesion efficacy in vivo was further analyzed quantitatively (Figure 9). The evaluated proximal interphalangeal (PIP) and distal interphalangeal (DIP) joint angles are physiologically relevant for assessing the range-of-motion of the joint and providing meaningful comparison regarding the seriousness of adhesion formation after different treatments. Compared with the control group, the joint flexion DIP angle of the HAIFB group increased to the value of the normal FDP tendon (dotted line) and showed statistical improvement compared with the control group 3 weeks post-operation (Figure 9A). Although an increasing trend of DIP angles were found for Seprafilm^®^ and HAFB groups, they fell short of the normal FDP tendon value and only the HAFB group showed statistical improvement of DIP angle from the control group. Similar to the trend observed for the DIP angle, the PIP angle of the HAIFB group was the highest, crossing the normal tendon angle value and significantly higher than all other groups. Indeed, the overall trend of average joint angles indicated tendons treated with HAIFB will display better range-of-motion than HAFB, which was further better than Seprafilm^®^ and the control. The higher DIP and PIP angle values, which were close to those of the normal tendon, indicated the recovery of range-of-motion for the injured tendon 3 weeks after treatment with HAIFB, which could prevent post-surgical peritendinous adhesion in vivo [54,55].

From the tendon gliding experiment, the gliding excursion distance revealed the same trend as the joint range-of-motion DIP and PIP angles in Figure 9A,B, i.e., HAIFB showed the least adhesion with the highest excursion, followed by HAFB and Seprafilm^®^, while the control was the most adhesive with the least excursion distance (Figure 9C). The gliding distance of HAFB was significantly higher than the control and Seprafilm^®^, with no difference among the two. Therefore, the tendon gliding experiment clearly indicated that the gliding excursion of the HAIFB group was significantly higher than the other groups and similar to the normal tendon. Since adhesion severity could be judged from the difference in the gliding displacement value, the excursion distance from the tendon gliding experiments further demonstrated the efficacy of IBU-loaded NFMs (HAIFB) in preventing peritendinous adhesion.

The pull-out force, which is defined as the required force to completely remove the FDP tendon from the tendon sheath by pulling, was also determined. As more severe adhesion will lead to higher pull-out force, it showed a reverse trend from previous range-of-motion biomechanical tests, with the untreated control group requiring the maximum force (10.1 N) to remove the FDP tendon from the tendon sheath due to the fact of severe adhesion (Figure 9D). The tendon treated with Seprafilm^®^ needed the second largest pull-out force (5.3 N), while the HAFB group required 3.6 N to remove the tendon from the sheath. In accordance with previous anti-adhesion biomechanical testing, a repaired tendon covered with HAIFB showed the least adhesion judging from its minimum pull-out force (2.5 N), which was significantly lower than all other groups and approached that of a normal tendon (dotted line) [6].

Taken together, the biomechanical evaluation indicated that the repaired tendon wrapped with the HAIFB NFM post-surgically will result in the most favorable anti-adhesion outcome with following anti-adhesion efficacy HAIFB > HAFB > Seprafilm^®^ > control. This is consistent with the gross observation and H&E staining results shown in Figure 8A–H. As a less inflammatory response of the healed tendon at the surgical site was found for IBU-loaded HAIFB NFMs from F4/80 and TNF-α IHC staining (Figure 8L,P), we successfully correlated its preference over NFMs without IBU (HAFB) to lower tendon adhesion with smaller pull-out force, higher DIP/PIP angles, and higher gliding excursion from biomechanical testing (Figure 9). This improvement in anti-adhesion efficacy should be offered from the prompt release of IBU from HAI30FB NFMs (Figure 4) without eliciting cytotoxicity (Figure 5).

## 3. Materials and Methods

### 3.1. Materials

Hyaluronic acid (HA; sodium hyaluronate; average molecular weight = 1.3 × 10^6^ Da; range of molecular weights = 0.8–1.5 × 10^6^ Da) was purchased from Shandong Freda Biochem (Jinan, China); ibuprofen (IBU), poly(ethylene oxide) (PEO; average molecular weight = 2.0 × 10^6^ Da; viscosity = 2000–4000 cP for 2% solution in water at 25 °C), iron (III) chloride (FeCl_3_) hexahydrate, and 1,4-butanediol diglycidyl ether (BDDE) were purchased from Sigma–Aldrich (St. Louis, MO, USA). Antibiotics and trypsin-EDTA were obtained from Thermo Fisher Scientific (Waltham, MA, USA). CellTiter 96^®^AQueous One solution was purchased from Promega (Madison, WI, USA). Dulbecco’s modified Eagle’s medium (DMEM) and fetal bovine serum (FBS) were obtained from Life Technologies (Carlsbad, CA, USA). The actin cytoskeleton and focal adhesion staining kit (FAK100) was purchased from Millipore (Bedford, MA, USA).

### 3.2. Fabrication of Nanofibrous Membranes (NFMs)

The HA/IBU/PEO solutions were prepared separately by mixing 0.0563 g, 0.0964 g, and 0.1500 g of IBU with 0.175 g of HA and 0.050 g of PEO in 10 mL of formic acid solution to obtain 20%, 30%, and 40% (*w*/*w*) IBU polymer solutions for preparation of HAI20, HAI30, and HAI40 NFMs. An electrospinning setup was used, comprising a 23 gauge stainless-steel needle, a 5 mL glass syringe, and a syringe pump (KD Scientific, Holliston, MA, USA). The syringe delivered the polymer solution at 1 mL/h by connecting with a high-voltage DC power supply (Glassman High Voltage Inc., High Bridge, NJ, USA) to the needle tip, which provided a 20 kV voltage. An NFM with an approximate thickness of 200 μm was collected on a grounded aluminum target that was placed 15 cm away from the needle tip. To prepare the iron ion-crosslinked NFMs, the solution immersion method was used with some modification [56]. Briefly, a 6 cm × 5 cm HAI20, HAI30 or HAI40 NFM was immersed in a 30% (*w*/*w*) FeCl_3_ solution in anhydrous ethanol in a 10 cm diameter Petri dish and shaken at 30 rpm for 24 h to obtain HAI20F, HAI30F and HAI40F NFMs. Each membrane was washed with 10 mL of anhydrous ethanol twice to remove residual Fe^3+^ ions and vacuum dried for 24 h to get ionically crosslinked HAIF NFMs with different IBU contents. The ionically crosslinked HAI20F, HAI30F, and HAI40F NFMs were further separately reacted with 10% (*w*/*w*) of BDDE in anhydrous ethanol in a 10 cm Petri dish and shaken at 30 rpm for 24 h to produce HAI20FB, HAI30FB, and HAI40FB NFMs. The membrane was washed with 10 mL of anhydrous ethanol twice to remove residual BDDE and vacuum dried for 24 h to get dual crosslinked HAIFB NFMs. For comparison, HAFB NFMs were prepared following the same procedure but using HA/PEO solutions without IBU.

### 3.3. Characteristics of Nanofibrous Membranes (NFMs)

The surface morphology of HAIFB NFMs was observed using a scanning electron microscope (SEM, Hitachi S3000N, Tokyo, Japan). At least 100 fibers randomly chosen from 10 images were used to measure the fiber diameter with the ImageJ software. The thermal properties of the NFMs were evaluated using thermogravimetric analysis (TGA) and derivative thermogravimetry (DTG) (TGA 2050, TA Instruments, New Castle, DE, USA) up to 600 °C at a heating rate of 10 °C/min. For mechanical properties, a material testing machine (Tinius Olsen H1KT, Horsham, PA, USA) equipped with a 10 N load cell was used to measure the uniaxial tensile properties of NFMs by operating at 5 mm/min elongation rate. A 1 cm × 5 cm test membrane sample was vertically mounted on two mechanical gripping units at each end, leaving a 3 cm gauge length for mechanical loading. The ultimate tensile stress, ultimate tensile strain, and Young’s modulus were obtained from the stress–strain curve. Chemical analysis was performed using attenuated total reflection Fourier transform infrared (ATR-FTIR) spectroscopy from 400 to 4000 cm^−1^ with 2 cm^−1^ resolution using a Horiba FT-730 spectrometer (Kyoto, Japan) and recorded as transmittance versus wavenumber. To determine the release of IBU, the NFMs were punched into disc-shaped membranes (1.5 cm in diameter) and sterilized by UV exposure for 2 h in an UVP CL-1000 Crosslinker at 0.1 J/cm^2^ for 2 h. Sterilized NFMs were immersed in 4 mL of phosphate buffered saline (PBS, pH = 7.4) in a vial and placed in a shaking incubator at 37 °C. After a fixed time, the whole solution was removed from the vial, followed by replenishing with 4 mL of PBS to the vial to continue the drug release experiments. To measure IBU concentration in the solution, high-performance liquid chromatography (HPLC) was used with a Hypersil^™^ ODS C18 column (5 μm) using 55%/45% methanol/water as the mobile phase at 1 mL/min flow rate and detected with an UV detector at 254 nm. The percentage of released IBU was calculated based on the amount of IBU used in preparing the NFM and presented in terms of cumulative release percentage from cumulative amount of released IBU.

### 3.4. In Vitro Cell Culture Studies

#### 3.4.1. Cell Penetration through NFMs

The NIH/3T3 mouse embryonic fibroblast cells (3T3) (ATCCCRL1658) were purchased from American Type Culture Collection (Manassas, VA, USA). The barrier effect of HAIFB NFMs was evaluated using the cell penetration test developed previously in our group [47]. The 3T3 cells were cultured in a double chamber dish containing DMEM with 2% or 10% FBS and divided with a porous membrane (Transwell cell culture insert). The concentration of FBS was 2% in the upper chamber and 10% in the lower chamber. An NFM was placed at the bottom of the cell insert and the 3T3 cells were inoculated at a density of 1 × 10^5^ per well in the upper chamber. Control experiments were conducted without placing an NFM at the bottom of the cell culture insert. To estimate the number of viable cells penetrated into the lower chamber after 24 h, an MTS cell viability assay with the CellTiter 96^®^AQueous One solution was used, which contains a novel tetrazolium salt to interact with metabolically active cells and produce a soluble formazan dye for detection at 492 nm. The optical density value (OD_492_) was determined using an ELISA plate reader (BioTek Synergy HT, Winooski, VT, USA) and converted to viable cell numbers using a standard curve. Qualitative evaluation of penetrated cells was also verified by observing the lower chamber surface under an inverted microscope (Olympus IX71, Tokyo, Japan).

#### 3.4.2. Cell Attachment and Proliferation

The NFMs were cut into 1.5 cm disc-shaped membranes and sterilized by UV light exposure for 2 h before use. The NFMs were placed in a 24 well culture plate, seeding with 0.2 mL of 3T3 cell suspension at a seeding density of 1 × 10^4^ cells per well and incubating at 37 °C for 4 h for cell adhesion. Subsequently, 1.5 mL of cell culture medium (DMEM containing 10% (*v*/*v*) FBS and 1% (*v*/*v*) antibiotic-antimycotic) was added to each well and maintained at 37 °C in a humidified 5% CO_2_ incubator. The number of cells attached to an NFM was determined on day 1 and 7 from DNA assays using Hoechst 33258. Cells cultured on tissue culture polystyrene (TCPS) surface were used for comparison.

#### 3.4.3. Cytoskeleton and Focal Adhesion Analyses by Confocal Microscopy

The cytoskeletal arrangement and focal adhesion of attached 3T3 cells on NFM were analyzed using an actin cytoskeleton/focal adhesion staining kit. After cultured in cell culture medium for 1 day, the cells were washed in PBS and fixed in 4% paraformaldehyde solution for 20 min. Further treatment with 0.1% Triton X-100 for 10 min at room temperature was used to permeabilize the cells. For cell staining, the cells were incubated with tetramethylrhodamine (TRITC)-conjugated phalloidin for 30 min for actin filaments, mouse anti-vinculin primary antibody (1 h), and fluorescein isothiocyanate (FITC) AffiniPure goat anti-mouse IgG secondary antibody (1 h) for vinculin focal adhesion protein. Next, 4′, 6-diamidino-2-phenylindole (DAPI) was used to stain the nucleus for 5 min. A confocal laser scanning microscope (Zeiss LSM 510 Meta, Oberkochen, Germany) was used to visualize the fluorescence signal at excitation/emission wavelength of 540 nm/545 nm (red), 340 nm/488 nm (blue), and 528 nm/617 nm (green), which represent the stained actin cytoskeleton, nucleus, and vinculin, respectively. Cells cultured on TCPS were used as control for comparison.

### 3.5. Animal Studies

#### 3.5.1. Flexor Tendon Adhesion Animal Model

The animal study was approved by the Institutional Animal Care and Use Committee (IACUC) of Chang Gung University (IACUC Approval No.: CGU14-078, approved on 9/22/2014). New Zealand white rabbits (12 weeks) were purchased from the National Laboratory Animal Breeding and Research Center (Taipei, Taiwan). The skin of the hindpaw was shaved and sterilized after the induction of general anesthesia through intramuscular injections of xylazine (6.7 mg/kg body weight) and ketamine (33.3 mg/kg body weight). The zone-II flexor tendons of the second and third digits from each hindpaw of the rabbits were released from the tendon sheath. The flexor digitorum superficialis (FDS) tendons were removed first and the flexor digitorum profundus (FDP) tendons were cut completely with a scalpel, just distal to the chiasm and proximal to the vincula. The FDP tendons were repaired with modified Kessler core suture technique using 5-0 braided polyester sutures. The animals were randomly divided into four groups (control, Seprafilm^®^, HAFB NFMs, and HAI30FB NFMs). All NFMs were sterilized with ethylene oxide before use. For each experimental group, a 10 mm × 12 mm piece of Seprafilm^®^, HAFB or HAI30FB was wrapped around the tendon repair site, whereas PBS solution was poured on the tendon repair surface in the control group. The skin was closed with 5-0 Nylon sutures and the operated leg was immobilized in a cast to limit interphalangeal joint movements. The animals were allowed unrestricted activity and received food and water ad libitum. After three weeks, animals from each group were euthanized using lethal doses of pentobarbital (0.5 g/kg body weight) and the toes were transected at the metatarsophalangeal joints. The skin incisions were reopened through the original suture line and the digits were randomly assigned for evaluation of peritendinous adhesions. Assessments were performed through gross evaluation, histological analysis, range-of-motion (flexion angle) of the distal interphalangeal (DIP) and proximal interphalangeal (PIP) joints, tendon gliding excursion (distance), and tendon pull-out force [24].

#### 3.5.2. Gross Observation

A midline incision was made at the plantar side of the experimental toe to expose the repaired FDP tendon. A macroscopic adhesion grading system was implemented to estimate the severity of adhesion around the repaired tendon by two surgeons who were blinded to the groups. The grading system was as follows: grade 0, no marked adhesion; grade 1, filmy adhesion that could be readily separated using blunt dissection; grade 2, mild adhesion; grade 3, severe adhesion [25,26].

#### 3.5.3. Histological Evaluation

The second and third digits of the rabbits were harvested for histological evaluations. Subsequently, the specimens were fixed in 10% formaldehyde in PBS, sectioned into 5 μm slices and stained with hematoxylin and eosin (H&E) using standard protocols. For immunohistochemical (IHC) staining of F4/80 and tumor necrosis factor alpha (TNF-α), mouse anti-TNF-α (Novus NBP2-34301, Centennial, CO, USA) and rabbit anti-F4/80 antibody (Abcam ab240946, Cambridge, UK) were used as the primary antibody. The ImmPRESS Excel Amplified HRP Polymer Staining Kit (Thermo Fisher Scientific, Waltham, MA, USA) containing the goat anti-mouse IgG or goat anti-rabbit IgG Amplifier Antibody, the ImmPRESS Excel Amplified HRP Polymer Reagent (anti-goat IgG), and the ImmPACT DAB EqV Substrate was used. The stained sections were examined under an Olympus IX-71 inverted microscope.

#### 3.5.4. Biomechanical Evaluation

A range-of-motion test of toe joint flexion and the length of FDP tendon gliding distance were performed by transecting the sample at the same point at the proximal metacarpal level, suturing to a cable, and connecting to a load transducer of a custom-made range-of-motion device. The metatarsophalangeal joint was fixed by inserting a wire longitudinally through the metatarsal and the proximal phalanx. The proximal, middle, and distal phalanges were fixed to T-shaped pins containing two reflective markers. Subsequently, the prepared digit was mounted on the range-of-motion device by fixing the proximal wire to a non-slip clamp. A 50 g weight was attached to the extensor tendon to apply an initial tension and ensure full extension of the digit. The actuator pulled the tendon slowly at a rate of 3 mm/s to cause digital flexion (angular range of motion). The angle measured between the distal and middle phalanges determined the DIP joint flexion, and the angle between the middle and proximal phalanges determined the PIP joint flexion [27]. For the functional evaluation based on tendon gliding excursion, two metal pins were inserted through the proximal phalanx to fix the digits to a table, and then traction was applied to the tendon, thus flexion of the distal joints could easily be performed. The FDP tendon was exposed by removing the skin, subcutaneous tissue, and the other flexor tendons. The tendon sheath and FDP tendon were marked at the exit from the sheath, and a counterweight from the distal phalanx was applied to fully extend the interphalangeal joints. A constant force of 1 N was applied to pull the FDP tendon out of the sheath tunnel, and the distance after pulling was measured with a micrometer caliper. This tendon gliding distance was recorded as the gliding excursion of the FDP tendon. To evaluate peritendinous adhesions, the pull-out force was measured using a material testing machine (Tinius Olsen H1KT, Horsham, PA, USA) with a 50 N load cell. The digit was amputated through the metatarsophalangeal joint, leaving a 2 cm tail FDP tendon firmly fixed at the bottom by a custom-made static clamp. The distal digit was attached to the hook through a hole drilled in the distal phalanx and connected through a steel hook to the top crosshead. The FDP tendon was pulled at a rate of 5 mm/min and, ultimately, the movement of the crosshead pulled the tendon out of the tendon sheath. The pull-out force (N) was calculated by measuring the maximum force necessary to pull the tendon out of the tendon sheath.

### 3.6. Statistical Analysis

The results are expressed as mean ± standard deviation. SPSS 10.0 (Chicago, IL, USA) was used to analyze the data with a one-way analysis of variance (ANOVA) with *p* < 0.05 being considered significant.

## 4. Conclusions

In this study, electrospinning was used successfully to prepare NFMs loaded with 20 to 40% IBU (HAIFB) for controlled release of the anti-inflammatory drug in reducing postoperative tendon inflammation and preventing tendon adhesion. The microporous structure and excellent mechanical properties of the NFM enables proper tendon healing while providing a powerful physical barrier to prevent postoperative fibroblastic penetration, which leads to tissue adhesion. The different loading content of IBU did not change the macroporous nature of the NFMs, which contained nanofibers with similar fiber diameter and physico-chemical properties. However, higher IBU loading improved the mechanical properties of the NFMs, with higher ultimate stress (strain) and modulus. From in vitro cell culture experiments with fibroblasts, HAIFB NFMs could prevent cell attachment and penetration, which in turn could reduce the formation of tissue adhesions in vivo. Nonetheless, IBU loading above 30% induced cytotoxicity stemming from a higher concentration of released IBU. From an in vivo study using a rabbit flexor tendon rupture animal model, we confirmed that IBU-loaded HAIFB NFMs (HAI30FB) are an ideal physical barrier membrane to simultaneously inhibit inflammation and tendon adhesion formation. The HAI30FB membrane exhibited excellent anti-adhesion efficacy over Seprafilm^®^ and non-IBU-loaded NFMs (HAFB) from gross observations, histological analyses, and biomechanical testing evaluations.

## Figures and Tables

**Table 1:** The ultimate tensile stress, ultimate tensile strain, and Young’s modulus of HAIFB NFMs with 20% (HAI20FB), 30% (HAI30FB), and 40% IBU (HAI40FB) loading.

Membrane	Ultimate Stress (MPa)	Ultimate Strain (%)	Young’s Modulus
HAI20FB	0.63 ± 0.53	61.46 ± 11.42	9.42 ± 0.83
HAI30FB	0.94 ± 0.89 *	81.22 ± 8.23 *	10.57 ± 0.84
HAI40FB	1.43 ± 0.13 *^, #^	90.11 ± 8.75 *^, #^	14.16 ± 1.25 *^, #^

* *p* < 0.05 compared with HAI20FB, ^#^ *p* < 0.05 compared with HAI30FB.

## Abbreviations

ATR-FTIR	Attenuated total reflection Fourier-transform infrared
BDDE	1,4-butanediol diglycidyl
DMEM	Dulbecco’s modified Eagle medium
DTG	Derivative thermogravimetry
DAPI	4′,6-diamidino-2-phenylindole
DIP	Distal interphalangeal
ELISA	Enzyme-linked immunosorbent assay
FDP	Flexor digitorum profundus
FDS	Flexor digitorum superficialis
FBS	Fetal bovine serum
FITC	Fluorescein isothiocyanate
GMP	Good manufacturing practice
HA	Hyaluronic acid
H&E	Hematoxylin and eosin
IBU	Ibuprofen
IHC	Immunohistochemical
NFM	Nanofibrous membrane
NSAIDs	Nonsteroidal anti-inflammatory drugs
OD	Optical density
PBS	Phosphate buffered saline
PCL	Poly(caprolactone)
PEG	Poly(ethylene glycol)
PEO	Poly(ethylene oxide)
PLA	Poly(lactic acid)
PIP	Proximal interphalangeal
PIP	Proximal interphalangeal
SEM	Scanning electron microscopy
TGA	Thermogravimetric analysis
TCPS	Tissue culture polystyrene
TNF-α	Tumor necrosis factor alpha
